# Women's Mental Health in the Time of Covid-19 Pandemic

**DOI:** 10.3389/fgwh.2020.588372

**Published:** 2020-12-08

**Authors:** Florence Thibaut, Patricia J. M. van Wijngaarden-Cremers

**Affiliations:** ^1^Department of Psychiatry, University of Paris, University Hospital Cochin-APHP, INSERM U1266, Institute of Psychiatry and Neurosciences, Paris, France; ^2^Department of Addiction and Developmental Disorders, Dimence GGz Zwolle, UMCN St Radboud, Nijmegen, Netherlands

**Keywords:** domestic violence, gender, women, mental health, Covid-19, pregnancy, pandemic

## Abstract

Even if the fatality rate has been twice higher for men than for women, the Covid-19 pandemic has affected women more than men, both as frontline workers and at home. The aim of our article was to analyze the differences observed in mental health and violence between men and women in the COVID outbreak. For this purpose, we have used all papers available in PubMed between January and July 2020 as well as data from non-governmental associations. We have thus successively analyzed the situation of pregnancy during the pandemic; the specific psychological and psychiatric risks faced by women both as patients and as workers in the health sector, the increased risk of violence against women at home and at workplace and, finally the risk run by children within their families. In conclusion, research on the subject of mental health issues during the Covid-19 pandemic is still scarce, especially in women. We hope that this pandemic will help to recognize the major role of women at home and at the workplace.

## Introduction

The Covid-19 outbreak is the most severe pandemic since the H1N1 influenza (Spanish flu) pandemic that occurred in 1918.

In Western Europe, men represented a slight majority of coronavirus cases (52–58%) but around 70% of coronavirus deaths. In contrast, in South Korea, men represented less cases of coronavirus cases (40%) but still a slight majority of coronavirus deaths (around 52%). The largest proportion of deaths (male-to-female ratio) in confirmed cases was observed in Myanmar, Thailand, Albania and Wales (ratios > 2) (September 2020) (https://globalhealth5050.org/the-sex-gender-and-covid-19-project/). The highest death ratio reported in men may be partly explained by pre-existing cardio-vascular or metabolic diseases, as well as a higher prevalence of at-risk behaviors such as alcohol abuse or tobacco smoking. Interestingly, according to Johnson et al. (1), women seem more likely to follow hand hygiene practices, which may decrease the infectious risk. In addition, sex chromosomes, sex hormones may contribute to the differences observed between males and females in the immune responses (2).

Yet, Covid-19 pandemic has affected women more profoundly than men in several areas, both at workplace (especially in the health and social sector), and at home with an increased workload due to lockdown and quarantine measures. Worldwide, 70 percent of the health workforce is made up of women who are often frontline health workers (nurses, midwives and community health workers). Similarly, most of health facility service-staff (cleaners, laundry, catering) is made up of women (3). In the US, women hold 78 percent of all hospital jobs, 70 percent of pharmacy jobs and 51 percent of grocery store roles (4). Consequently, women are more likely to be exposed to the virus (5). In Italy and Spain, 66 and 72% of health workers infected were female as compared with 34 and 28% of males respectively (3).

Many countries have reported an increase in domestic violence cases after the viral outbreak (6). Asking for more support with domestic burden can trigger domestic violence against women. In countries where lockdown is observed, home is unfortunately not always a safe space. The exacerbation of gender-based violence may not receive the attention needed in the context of the pandemic. Past experience from the Ebola and Zika epidemics have already shown that these crises have increased existing inequalities including those based on gender and economic status (UN issue-brief-covid-19-and-ending-violence-against-women-and-girls-en).

A lack of adequate domestic and emotional support can have consequences on women's mental health. The risk of anxiety, depression and post-traumatic stress disorder (PTSD) is also much higher in women (7, 8).

According to Mrs Phumzile Mlambo-Ngcuka, Executive director of United Nations (UN) Women: Covid-19 pandemic is not just a health issue, it is a profound shock to our societies exposing the deficiencies of public and private arrangements that currently function only if women play multiple and underpaid roles. This is a moment for governments to recognize both the enormity of the contribution women make and the precarity of so many (3).

The aim of this review was to analyze the differences observed in mental health and violence between men and women in the COVID outbreak. For this purpose we have used all papers available in PubMed between January and July 2020, using the following keywords: women, gender, pregnancy, domestic violence, mental health, pandemic and COVID-19; as well as data from non-governmental associations. The search was restricted to manuscripts written in English language and published in peer-reviewed journals. We have thus successively analyzed the situation of pregnancy during the pandemic; the specific psychological and psychiatric risks faced by women both as patients and as workers in the health sector, the increased risk of violence against women at home and at workplace and, finally the risk run by children within their families.

## The Specific Situation of Pregnancy During the Pandemic

### Infectious Risk

Several papers have reported a high rate of maternal and neonatal complications in COVID-19 positive pregnant women [(9, 10); for review see (11)]. According to a retrospective study conducted in the US by Lokken et al. (12), of 46 pregnant women SARS-CoV-2 positive nearly 15% developed severe Covid-19, which occurred primarily in overweight women with comorbid somatic disorders. However, the increased risk of having more severe COVID-19 disease during pregnancy, was not observed by Chen et al. (13).

COVID-19 was associated with a high rate of miscarriage, preterm birth, pre-eclampsia, cesarean (for unknow reasons), and perinatal death (14). However, Baud et al. (15) did not confirm the higher risk of miscarriage. A relatively high proportion of pregnant women (13.0%) were admitted to the intensive care unit, but no deaths were reported [(11): review of 13 Chinese studies]. In contrast, a study conducted on 116 Chinese pregnant women reported that no increased risk of spontaneous abortion and preterm birth was observed (16). Most of these studies were case reports or observational studies which may have contributed to these discrepancies. All these uncertainties are likely to increase the level of psychological stress and may contribute to an increased risk of pregnancy terminations.

As regard to the risk of neonatal infection, the proportion of infected neonates was low (6%) and two neonates died in Capobianco's review (11). In all cases, respiratory symptoms were observed. Interestingly, Vivanti et al. (17) described the first documented case of congenital COVID-19 infection associated with neurological symptoms following neonatal viremia. Transplacental transmission was associated with inflammation in the cerebral spinal fluid in the neonate and magnetic resonance imaging showed bilateral lesions of the white matter.

Although transmission of SARS- CoV-2 through breast milk was considered unlikely (18), some positive women may choose not to breastfeed to avoid direct contact with the newborn and reduce the risk of neonatal infection (19). Indeed, close contact of mother and infant after birth can increase the risk of transmission of the virus to the baby through droplets or micro-droplets. Sighaldeh et al. (20) recommended separating the baby from the mother with confirmed (or even suspected) COVID-19 infection for at least 2 weeks. In addition, infected mothers should be taught about the symptoms of baby's infection in case it happens, and the principles of hygiene to protect the baby and prevent transmission.

### Psychological Risk

The pandemic can be particularly distressing during specific situations such as pregnancy. In a Canadian study, two cohorts of pregnant volunteer women were compared (21). The first one was recruited before the COVID-19 pandemic (*n* = 496); the second one (*n* = 1,258) online during the pandemic in April 2020. This study was only focused on distress and psychiatric symptoms. Women from the COVID-19 cohort as compared with pre-COVID-19 women showed higher levels of depressive and anxiety symptoms (OR = 1.94). Moreover, in the COVID-19 cohort, women with previous psychiatric diagnosis or low income were at higher risk to report elevated distress and psychiatric symptoms.

### Potential Risk for the Children

Moreover, we do not know yet the after-effects of maternal exposure to COVID-19 infection and the risk of future mental disorders in offspring since the virus may have toxic effects on fetal brain. Vivanti et al. (17) reported the first case of a neonate with white matter injury due to a COVID-19 infection after transplacental transmission. At 2 months after birth, the neonate's hypertonia was improved and white matter lesions were reduced. However, early brain lesions may increase the risk of further mental disorders (22).

## Women May Be at Higher Risk of Psychiatric Symptoms During the Pandemic

In order to investigate the prevalence of psychiatric disorders during the COVID-19 pandemic peak, several large surveys were conducted online in the general population. Liu et al. (23) found a prevalence of post-traumatic stress symptoms of 7% in Wuhan (China) 1 month after the COVID-19 outbreak (in 285 residents). In sub-symptom analysis of PCL-5 (PTSD Checklist for DSM-5), women suffer more re-experiencing, negative alterations in cognition or mood and hyper-arousal as compared to men. In the same way, Li and Wang (24) found that 29.2% of the 15,530 respondents in the UK scored 4 or more on general psychiatric disorders measured by the 12-item General Health Questionnaire (GHQ-12) and 35.86% of the respondents sometimes or often feel lonely. Women and young people had higher risks of psychiatric disorders and loneliness. Being employed and living with a partner were protective factors. Moreover, participants who have or had COVID-19-related symptoms were more likely to have psychiatric disorders. Liu et al. (25) have also reported high levels of depression (43.3%, PHQ-8 scores ≥ 10), anxiety scores (45.4%, GAD-7 scores ≥ 10), and PTSD symptoms (31.8%, PCL-C scores ≥ 45) in 898 Americans (18–30 years) during the pandemic. In this latter study, no differences were observed between men and women.

## Health Care Workers (Especially Women) Were at Higher Risk of Mental Health Symptoms

The WHO postulated that many health care providers could develop PTSD, depression, anxiety and burnout during and after the pandemic peak (5). Lai et al. (26) have conducted a cross-sectional study in 1257 Chinese health care workers treating patients with COVID-19 (76.7% of all participants were women, and 60.8% were nurses). They found a high prevalence of mental health symptoms. In total, 50.4, 44.6, 34.0, and 71.5% of participants reported symptoms of depression, anxiety, insomnia, and more than 70% reported psychological distress, respectively. Female gender and having an intermediate occupation were associated with experiencing more severe depression, anxiety, and distress. Working as a frontline health worker (41.5% of the participants) and in Wuhan (the epicenter of the crisis) were also risk factors for worse mental health outcomes. In fact, the chance of being infected was much higher in this latter group, which added a fear of transmission to their families. In contrast, Chew et al. (27) reported that in 906 healthcare workers (64.3% were female) from Singapour and India, only 5.3% had moderate to severe depression, 8.7% had moderate to severe anxiety, 2.2% moderate to severe stress, and 3.8% moderate to severe levels of psychological distress. The most common symptom observed was headache (32.3%). A significant association between the prevalence of physical symptoms and psychological outcomes was reported but no association was observed with gender. As a comparison, 10% of 549 health care workers reported high levels of PTSD symptoms at some time during the 3 years following the severe acute respiratory syndrome (SARS) epidemic in 2003 (28). Being single and with low income were risk factors for PTSD. In another study conducted during the previous SARS pandemic in Hong Kong, 25% of health care workers required psychological follow up (29). Gender was not taken into account in these analyses.

Furthermore, the conflict professionalism as well as personal fear for one's health contributed to burnouts as well as physical and mental symptoms in health workers (30). Increased workload, isolation, and discrimination were also common in caregivers and could result in physical exhaustion, fear, emotional disturbance, and sleep disorders (31). In addition, in the time of pandemic, few adequate services may screen physicians and nurses in contact with infected patients for anxiety, depression and suicidality and provide counseling.

## Psychiatric Symptoms in COVID 19 Positive Patients

Guo et al. (32) reported that COVID-19 positive patients had higher levels of depression, anxiety, and post-traumatic stress symptoms as compared with normal controls. Women reported significantly more “Perceived Helplessness” as compared to men and controls. There was a correlation between depression and CRP levels among patients indicating that the immune-inflammatory response may be involved. Many patients complain also of intense fatigue and apathy in the weeks or months following infection, which have already been observed with previous SRAS infections or influenza. These symptoms highlight the link between depression, viral infections and inflammatory mechanisms (33). Further exploration of the mental health outcome of COVID-19 positive patients using an gendered lens would be of high interest.

Similarly, studies exploring the psychological consequences of the 2002–2004 SARS outbreak in China reported that anxiety and depression as well as PTSD occurred after the epidemic. At 30 months post-SARS, 25 percent of the patients had PTSD, and 15.6 percent depressive disorders (34). Mak et al. (35) and Lam et al. (36) reported more that 40% of SARS survivors had post traumatic stress symptoms (PTSS). Single subjects, those working in high-risk workplaces, or having close relatives with SARS were two to three times more likely to develop high levels of PTSS than those not exposed to the virus (37).

## Domestic Violence

Intimate partner violence (IPV) includes physical or sexual violence, emotional abuse and stalking. It is the major cause of homicide death for women (38). Victims of IPV are at increased risk of multiple mental disorders as well as somatic diseases (cardiovascular disease, chronic pain, sleep disturbances, gastrointestinal problems, sexually transmitted infections, traumatic brain injury) (39). Exposure of children to family violence may also increase the risk of perpetrating violence in their adult relationships (40). Several risk factors have been identified: low income, social isolation, loss of bearings, narrowness of premises, loss of loved ones, fear of dying, difficulties in accessing medical and social services, inability to flee, increased consumption of addictive substances, etc. (41–44). All these risk factors usually associated with intra-family violence are increased during epidemics. In addition, male aggression with or without alcohol often appears as a mode of reaction to a crisis (45). In these situations of dramatic crises, male aggression has long been more easily excused, especially when the anger was only temporary and had been the subject of sincere regret. Male violence may even have seemed legitimate for some people, as at it can be normal for a man to behave aggressively in situations of crisis and personal suffering, women then are being accused of having over-reacted or their requests for help in the face of violence have sometimes been simply ignored (46). For women at high risk of abuse, home may not be a safe place. Without private place, many women will find difficult to make a call or to seek help online. Similarly, in all crisis situations, whether wars, natural disasters or serious epidemics, whatever the country concerned, intra-family violence increases. In the aftermath of Hurricane Katrina, which occurred in 2009 in the United States, the prevalence of domestic violence had quadrupled; the physical violence suffered by women had almost doubled (4.2 to 8.3%) but remained unchanged for men (47). In the weekend following the 2010 New Zealand earthquake, police reported a 50 percent increase in calls for family violence (48). Pregnant women are also not immune to physical violence since after the Fukushima disaster, physical violence against pregnant women was four times greater in this region compared to other Japanese provinces at the same time, which was approximately 1.5 percent (49). In the same way, data from Hubei province in China, particularly affected by the coronavirus epidemic, showed a tripling of reports of intrafamilial violence in February 2020 during confinement compared with February 2019 (50). In the UK, a project tracking violence against women reported that deaths from domestic abuse between 23 March and 12 April had more than doubled (16 deaths) compared with the average rate in the previous 10 years (51). There are many reports of increased violence against women worldwide, with increases of 25 to 30% in countries with reporting systems (6, 52). These figures may reflect only the worst cases. More complex forms of violence may also develop when perpetrators may further restrict access to services and psychosocial support. Exposure to COVID-19 can be used as a threat. Abusers can also exploit the inability of women to call for help or escape; women may even be put out on the street without any shelter (43).

The disruption of protective networks may further exacerbate IPV and its consequences. The reduced functioning of the justice services and the fear of contamination in prisons make it more difficult to manage the perpetrators. Police and health services, which are the first line responders are overwhelmed and less available. Support services are affected by lockdown or, in some cases, reallocation of resources. Domestic violence shelters may be full, closed or repurposed. Yet, domestic violence shelters must remain open during the lockdown. UN Women Policy Brief (3) has reported some examples of how the government can help during the pandemic: in China, the hashtag #AntiDomesticViolenceDuringEpidemic has links to online resources; free calls to helplines were implemented in Antigua and Barbuda; in Spain, an instant messaging service with a geolocation function offers an online chat room with immediate psychological support; in the Canary Islands, Spain, and France, women can alert pharmacies about a domestic violence situation with a code message “Mask-19” that warns the police; in the UK police has enlisted postal workers and delivery drivers who can look out for signs of abuse. A popular app called “Bright Sky” provides support and information, but can be disguised when the partners check the phones. In France, 20,000 hotel room nights were available to women needing shelter to escape from abusive situations; in Colombia, the government has guaranteed continued access to services, including legal advice, psychosocial advice, police and justice services, including hearings. Similarly, virtual justice system were established in different countries.

According to diverse media sources and women rights experts, different forms of online violence, such as stalking, bullying, sexual harassment, and sex trolling, have also increased during the pandemic (53).

Finally, reports of both physical and verbal attacks on healthcare workers have increased in China, Italy, France, and Singapore (54). Given the higher vulnerability of female frontline workers and the increased risk of violence against them, specific measures must be put in place to protect them.

## Violence Against Children: Type and Risk Factors

The number of calls to 119 for child victims of violence also increased by 20% with an increase in urgent calls by 60% compared to March 2019 in France. With regard to violence against children, low-income is the most often reported risk factor of violence against children; sexual violence being more likely against girls than boys (55). Other risk factors are past history of exposure to violence in parents, parental substance abuse, child labor (56). The closure of schools increased the risk of violence against children. Additional constraints faced by families as a result of the Covid-19 crisis such as job loss or falling income, social isolation, excessive confinement in often cramped premises, fear related to the pandemic situation, enhanced the risk of domestic violence, whether inflicted between partners or on children by adults who care for them (57, 58).

Simultaneously, the Covid-19 crisis increased the risk of child sexual exploitation on the internet. Europol recently reported that law enforcement auxiliaries are reporting more online activity by people looking for content from child abuse. The French government, in partnership with care services, victim assistance services and the justice system, has taken a certain number of measures to maintain assistance to victims during this period of confinement. Psychiatrists, like all doctors and health personnel, are on the front line in detecting violence against children (for example: https://www.stopblues.fr/fr/node/449).

## Conclusion

Research focused on the subject of mental health issues during the COVID-19 pandemic is still scarce, especially in women. Yet, Covid-19 pandemic has affected women much more profoundly than men, both as frontline workers and at home. Financial crisis is gradually developing and as a consequence mental health issues are likely to grow exponentially. According to the United Nations (3), women aged 24 to 34 are already 25% more likely than men to face extreme poverty.

Nevertheless, we should consider this pandemic as an opportunity to build better, stronger, more resilient societies that could bring relief as well as hope to all women on earth. For example, during the First World War and the concomitant flu pandemic, for the first time in the history of the United States, black nurses had the opportunity to serve the US army. In fact, this drama has been turned into an opportunity to improve gender equality (59). We hope that this pandemic will also help to recognize the major role of women at home and at the workplace. To achieve this goal, the UN recommended allocating additional resources to protect women, putting women at the center of policy changes and collecting more sex-disaggregated data to analyze the impact of pandemics on women (60). Moreover, the 17 Sustainable Development Goals (SDGs) proposed by the UN offer a unique opportunity to achieve gender equality (Goal number 5), which is a key element of all SDGs and simultaneously improve health and well-being for all before 2030 (61).

## Author Contributions

All authors listed have made a substantial, direct and intellectual contribution to the work, and approved it for publication.

## Conflict of Interest

FT is Editor-in-Chief of Dialogues in Clinical Neuroscience (the journal receives a grant from La conférence Hippocrate-Servier). The remaining author declares that the research was conducted in the absence of any commercial or financial relationships that could be construed as a potential conflict of interest.
